# An Overview of Traditional Uses, Phytochemical Compositions and Biological Activities of Edible Fruits of European and Asian *Cornus* Species

**DOI:** 10.3390/foods11091240

**Published:** 2022-04-26

**Authors:** Maria C. Tenuta, Brigitte Deguin, Monica R. Loizzo, Claire Cuyamendous, Marco Bonesi, Vincenzo Sicari, Lorenza Trabalzini, Anne-Claire Mitaine-Offer, Jianbo Xiao, Rosa Tundis

**Affiliations:** 1 Department of Pharmacy, Health and Nutritional Sciences, University of Calabria, 87036 Rende, Italy; mary.tn2006@hotmail.it (M.C.T.); monica_rosa.loizzo@unical.it (M.R.L.); marco.bonesi@unical.it (M.B.); rosa.tundis@unical.it (R.T.); 2 Faculté de Pharmacie de Paris, Université Paris Cité, U.M.R. n°8038-CiTCoM-(CNRS, Université de Paris Cité), F-75006 Paris, France; claire.cuyamendous@u-paris.fr; 3 Department of Agraria, “Mediterranea” University of Reggio Calabria, 89124 Reggio Calabria, Italy; vincenzo.sicari@unirc.it; 4 Department of Biotechnology, Chemistry and Pharmacy, University of Siena, 53100 Siena, Italy; lorenza.trabalzini@unisi.it; 5 Centre des Sciences du Goût et de l’Alimentation, CNRS, INRAE, Institut Agro, Laboratoire de Pharmacognosie, UFR des Sciences de Santé, Université de Bourgogne Franche-Comté, BP 87900, CEDEX, F-21079 Dijon, France; anne-claire.offer@u-bourgogne.fr; 6 Nutrition and Bromatology Group, Analytical and Food Chemistry Department, Faculty of Food Science and Technology, Universidade de Vigo, Ourense Campus, E-32004 Ourense, Spain; jianboxiao@yahoo.com; 7 School of Food and Biological Engineering, Chengdu University, Chengdu 610106, China

**Keywords:** *Cornus*, fruits, flavonoids, iridoids, beneficial health properties

## Abstract

*Cornus* species are widely distributed in central and southern Europe, east Africa, southwest Asia, and America. Several species are known for edible fruits, especially *Cornus mas* and *Cornus officinalis*. These delicious fruits, characterized by their remarkable nutritional and biological values, are widely used in traditional medicine. In contrast to the other edible *Cornus* species, *C. mas* and *C. officinalis* are the most studied for which little information is available on the main phytochemicals and their biological activities. Fruits are characterised by several classes of secondary metabolites, such as flavonoids, phenolic acids, lignans, anthocyanins, tannins, triterpenoids, and iridoids. The available phytochemical data show that the different classes of metabolites have not been systematically studied. However, these edible species are all worthy of interest because similarities have been found. Thus, this review describes the traditional uses of *Cornus* species common in Europe and Asia, a detailed classification of the bioactive compounds that characterize the fruits, and their beneficial health effects. *Cornus* species are a rich source of phytochemicals with nutritional and functional properties that justify the growing interest in these berries, not only for applications in the food industry but also useful for their medicinal properties.

## 1. Introduction

The genus *Cornus* L. (Cornaceae) comprises about 65 species widely distributed in central and southern Europe, east Africa, southwest Asia, and America [1]. Among the edible species of the genus, *Cornus*, *Cornus mas* L., *C. officinalis* Sieb. et Zucc, *C. kousa* Hanse, and *C. controversa* Hemsl. have fruits widely consumed in Europe and Asia [2].

*C. mas* (cornelian cherry) is native to southern Europe and southwest Asia [3]. This plant comes from the foothills of the Caucasus, and from there, it spreads over Romania, Bulgaria, Italy, Turkey, and the inland European continent [4]. Ripe (cherry red or dark) fruits are used for fresh consumption or the production of marmalade, jam, yoghurt, compote, liquor, soup with rice, syrup, juice, and wine [5].

*C. sanguinea* L. (European dogwood, blood twig dogwood, or common dogwood) is a species widely distributed in the temperate regions of Europe [6] and the Caucasian region. It is a small tree with leaves with a characteristic dark red colour in senescence [7]. It can be found in most vegetation types but usually in different mixed temperate broad-leaved forests dominated by oak, lime, maple, ash, elm and hornbeam. Usually, this species is cultivated for ornamental purposes for its decorative flowers and colourful leaves. Dogwood berries are a non-toxic fruit with a tart flavour that makes them unpleasant for raw consumption. They are used in jams and juices.

*C. officinalis* (Asiatic dogwood, Japanese cornel or cornel dogwood) is diffused in eastern Asia, mainly in China, Korea, and Japan [8]. It is a deciduous tree that grows in the warm temperate zone but presents a specific cold resistance; it can temporarily grow in a −18 °C low-temperature zone. *C. officinalis* red berries are edible and picked after the summer. They have been used in traditional Chinese medicine for more than 2000 years [9].

*C. kousa* (Korean dogwood and Japanese dogwood) is a native plant of East Asia, mainly in Korea, China, and Japan. It is a small deciduous tree, and its fruits have been traditionally used in therapeutic medicine [10]. Among *Cornus* species, the largest fruits are *C. kousa* fruits; they have a sweet flavour and are sometimes used for wine production. Like *C. sanguinea*, *C. kousa* is also cultivated as an ornamental tree because it presents a high resistance to pests and diseases [11].

*C. controversa* (giant dogwood) is a deciduous tree native to Japanese, China and Korean temperate forests. It grows until 15 m tall, and its ripe fruits (purplish-red or bluish-black) are smaller compared with other *Cornus* species [11].

After describing botanically the edible Cornus species and their use in folk medicine, our study focuses on listing and comparing the compounds belonging to the main classes of bioactive secondary metabolites, the main biological activities and the involvement of certain compounds in the effects known to date.

## 2. Study Design

The available information on *Cornus* species was collected from the following scientific databases: PubMed, Web of Science, Reaxys, SciFinder, Scopus and Google Scholar or the databases of publishers. The search terms used for this review included “*Cornus*”, “*Cornus* species”, “*Cornus mas*”, “*Cornus sanguinea*”, “*Cornus officinalis*”, “*Cornus controversa*”, “*Cornus kousa*”, “traditional uses”, “chemical profile”, “flavonoids”, “iridoids”, “monoterpenes”, “triterpenes”, “tannins”, “fatty acids”, “biological activity”, and “toxicity”.

The interest in *Cornus* species as a rich source of compounds with nutritional and biological properties is demonstrated by numerous studies in the literature.

Herein, we have covered the period from January 2017 to March 2022. However, several interesting works published before 2017 were described to comment on the reported data critically and evaluate and compare the most promising investigated bioactivities and the newly identified constituents.

To find relevant studies, works were screened based on title and abstract. Only English language papers were included in this study to confine our search. Inclusion criteria were phytochemical studies; in vitro, ex vivo, and in vivo studies; and studies with or without proposals of the mechanisms of action.

## 3. Traditional Uses of *Cornus* Fruits

Fruits, leaves, and flowers of *C. mas* have been traditionally used for more than 1000 years to treat several diseases, including measles, digestion problems, rickets, anaemia, hepatitis A, and pyelonephritis diseases [4,12,13,14]. In particular, all edible *Cornus* fruits are used in folk medicine (Table 1). *C. mas* and *C. officinalis* fruits are widely used in Europe and Asia for their applications in traditional medicine. *C. mas* is used in Slovakia to treat fever, digestive disorders, and inflammation, and in Iran for the treatment of malaria, diarrhoea, inflammatory bowel disease, fever, kidney stones, urinary tract infections, and cancer [4,15,16,17,18]. In Turkish folk medicine, the berries of *C. mas* and *C. sanguinea* are used as remedies for gastrointestinal disorders [19,20,21].

In Chinese traditional medicine, *C. officinalis* is known as a tonic to protect and nourish the kidney and liver [8]. In addition, it is accepted in the Chinese Pharmacopoeia (2010), and it is recognised as a principal and active herb in many medical prescriptions [44]. It is used alone or in combination with other herbs to ameliorate the clinical symptoms of liver and kidney disorders. In fact, the symptoms caused by kidney and liver deficiency, such as dizziness, weakness of the waist and knees, and tinnitus, were cured with *C. officinalis* and other plants [43]. Moreover, it is used to treat urinary disease, cough, asthmatic problems, diabetes, night sweats, frequent urination, impotence and seminal emission [48]. *C. controversa* is used in Korea and China as an astringent and tonic [46]. In traditional Korean medicine, *C. kousa* is employed to treat diarrhoea and as a haemostatic agent [47].

In conclusion, the traditional uses of these *Cornus,* mainly by the oral route, are very diverse. Some indications are often found: gastrointestinal disorders, throat and pulmonary infections, and kidney diseases. However, *C. mas* appears to be used to treat a wider range of infections.

## 4. The Main Chemical Constituents of *Cornus* Fruits

Edible fruits of *Cornus* contain phenolic compounds [49] such as tannins [9], anthocyanins [11,50], flavonoids [50], lignans [51], and aromatic acids, and terpenoids such as iridoids [52] as the main bioactive compounds [53]. However, currently, not all these classes of bioactive metabolites have been systematically investigated in the fruits of edible plants.

*C. mas* fruits have been the subject of numerous phytochemical studies, and many of its compounds are described. The fruits are rich in flavonoids, iridoids, triterpenes and fatty acids.

The literature shows that the chemical composition of the fruits of *C. officinalis* has also been extensively studied. Similarly, flavonoids, tannins, and iridoids are the main chemical constituents of this species. In addition, the presence of organic acids, polysaccharides, sterols, phenylpropanoids, lignans, furans and mineral substances has also been described. Early work on the fruits of *C. kousa* concerned the identification of triterpene glycosides [54] and lignans [55,56].

Recently, a comprehensive chemical profile of anthocyanins, flavonoids and organic acids was published [57]. Currently, only a few studies have investigated the chemical composition of other *Cornus* species. The identification of flavonoids was performed in *C. sanguinea*, and a description of the anthocyanins in *C. controversa* was carried out.

### 4.1. Flavonoids and Proanthocyanidins

Among flavonoids, flavonol glycosides are the major constituents, mainly with kaempferol or quercetin as aglycones. Quercetin 3-*O*-glucuronide is the major constituent in the methanol extract of *C. mas* fruits [50]. Other flavonols have an aglycon, such as myricetin. Flavanonols such as aromadendrin and flavanones including naringenin 3-*O*-methylester 7,3′-dihydroxy-5,4′-dimethoxyflavanone, and 4-acetoxy-5,2′,4′,6′-*β*-pentahydroxy-3-methoxychalcones are specifically found in the fruits. Among proanthocyanidins and flavonoids, catechin is predominant in the hydroalcoholic fruit extract, followed by epicatechin and procyanidin B_2_ [49].

The literature revealed that few studies of the chemical profile of *C. sanguinea* drupes have been conducted. Popović et al. [20] reported the presence of several quercetin 3-*O*-glycosides in the methanolic extract of the fruits. These are essentially quercetin glycosides that differ in the nature of the sugar part. Quercetin 3-*O*-glucuronide, 3-*O*-glucoside, 3-*O*-galactoside, 3-*O*-rhamnoside and 3-*O*-rutinoside (rutin) were found [20].

A recent study identified other heterosides, quercetin 3-*O*-*β*-D-galactopyranoside-4-*O*-*β*-D-glucopyranoside, quercetin 3,4′-di-*O*-*β*-D-glucopyranoside, myricetin 3-*O*-*α*-L-arabinopyranoside-4-*O*-*β*-D-glucopyranoside, ampelopsin 3-*O*-*β*-D-glucopyranoside and isorhamnetin 3-*O*-*β*-D-glucuronopyranoside [58].

The flavonoids reported in *C. officinalis* have been characterised by naringenin, kaempferol and quercetin aglycons [59]. In addition, it should be noted that quercetin and kaempferol heterosides are numerous. Epicatechin 3-*O*-gallate is only found in *C. officinalis* fruits [60].

Similar to other *Cornus* species, fruits of *C. kousa* are characterised by quercetin derivatives (quercetin 3-*O*-galactoside, quercetin 3-*O*-xyloside and quercetin 3-*O*-glucoside) and by kaempferol and myricetin heterosides [57,61,62]. The principal flavonoids and proanthocyanidins found in *Cornus* fruits are reported in Table 2.

The concentration is, in order: delphinidin 3-*O*-glucoside > delphinidin 3-*O*-rutinoside > cyanidin 3-*O*-glucoside [11]. In the same study, *C. kousa* fruits are analysed.

In this species, high amounts of cyanidin 3-*O*-glucoside are found, followed by cyanidin 3-*O*-galactoside and very small quantities of delphinidin 3-*O*-glucoside [11]. A recent study reported pelargonidin 3-*O*-galactoside in *C. kousa* fruits ([57]).

Among *Cornus* fruits, the lowest amounts of anthocyanins were found in the fruits of *C. kousa*. [11]. The anthocyanins isolated in *Cornus* fruits are reported in Table 3.

### 4.2. Lignans

Lignans were studied only in *C. officinalis* and *C. kousa* fruits. They were found by analysing an aqueous extract of *C. officinalis* and a hydroalcoholic extract of *C. kousa*.

Once again, a study of the *C. officinalis* extract revealed a very large number of compounds. A total of 18 lignans were identified from the aqueous extract; among them were frequent compounds with a dihydrodiconiferous skeleton, pinoresinol, and isolariciresinol. However, it should be noted that officinalignan A and B are unusual derivatives of neolignan glucopyranoside, which have only been identified in this species. Officinalignan B has a dihydrobenzofuran skeleton with a galloyl group attached [51]. Similar compounds, namely dihydrodehydrodiconiferyl alcohol (+)-pinoresinol, (+)-lariciresinol, threo and erythreo guaiacylglycerol-*β*-coniferyl aldehyde ether, (+)-pinoresinol, (-)-balanophonin and cornuskoside A, have been identified in C. *kousa* [56,72]. Table 4 reports the main lignans identified in *Cornus* fruits.

### 4.3. Tannins

Analysis of the literature on iridoid plants showed that tannins are scarcely or not present in the fruits. Among the *Cornus* edible fruits, tannins have been reported only in *C. officinalis* and *C. mas* [73].

For *C. mas*, the determination of tannins in fruit extracts revealed their presence in small or medium quantities, and so far, no tannins have been isolated and identified [74,75]. However, it should be noted that ellagic acid, a marker of hydrolysable ellagitannins, has been described in fruits and that hydrolysable tannins, such as cornusiins, are numerous in the stones of these fruits [49].

While so far, no data concerning identifying tannins in *C. mas* fruits are available, many tannins have been isolated from *C. officinalis* fruits. Therefore, the richness of hydrolysable tannins in *C. officinalis* was considered a remarkable difference compared to *C. mas*. The main constituents are ellagitannins and gallotannins such as 7-*O*-galloyl-D-sedoheptulose [9,73,76,77,78]. Among these tannins, dimers and trimers have molecular weights sometimes higher than 1000 D, such as cornusiins A−F and camptothins A−B. It should be noted that cornusiins were first found in the fruits of *C. officinalis*, and that recently, some of these compounds have been found in the stones.

### 4.4. Acids and Phenolic Acids

Twelve organic acids, namely malic, maleic, malonic, oxalic, succinic, citric, isocitric, tartaric, fumaric, quinic, shikimic, and ursolic acid, are identified in *C. mas* fruits [65,69,75,79,80]. Malic acid, followed by tartaric acid and citric acid, are the principal acids reported in the acidified aqueous extract of *C. mas* fruits collected in Bosnia and Herzegovina [65]. Fruits of *C. mas* collected in the Czech Republic possessed citric acid as the major organic acid [67]. Thirteen phenolic acids have also been described. Ellagic acid together with chlorogenic and gallic acids are the predominant phenolic acids found in hydroalcoholic extracts of C. mas fruits collected in Serbia [49]. Conversely, fruits from the Czech Republic reported chlorogenic acid as the principal phenolic acid. Other phenolic acids, including neochlorogenic acid, *p*-coumaric acid, vanillic acid, and ferulic acid, are reported in the methanol extract of *C. mas* fruits [81,82].

The acids described in *C. officinalis* are numerous. There are seventeen organic acids, including eight triterpenoids and twelve phenolic acids. Methyl gallate and gallic acid have been identified in the methanol extract of *C. officinalis* [83]. Other phenolic acids and acids, namely 2-*O*-(4-hydroxybenzoyl)-2,4,6-trihydroxyphenyl-methylacetate, *p*-coumaric acid, caffeic acid, ellagic acid, 3,5-dihydroxybenzoic acid, 3-hydroxy-2,4-di-amino-pentanoic acid, tartaric acid, betulinic acid, butyl malic acid, protocatechuic acid, dimethylmalate, malic, and 2-butoxybutanedioic acid, were recognised in C. officinalis fruits [64,84]. A few years later, caftaric acid monomethyl ester [60], caffeoyltartaric acid dimethyl ester [85], 3,5-dihydroxy-2-(2-methoxy-2-oxoethyl) phenyl 4-hydroxybenzoate [8], succinic acid, and citric acid were added to the chemical composition of *C. officinalis* fruits [86].

In *C. kousa* fruits, triterpenoid acids are numerous, and seven are described (arjunolic, asiatic, 19-hydroxyasiatic, betulinic, corosolic, maslinic, oleanic, pimaric, tormentic, ursolic and 2*α*-hydroxylursolic acid) [47,87,88]. Five different organic acids (citric, fumaric, oxalic, shikimic and malic acid) and three phenolic acids (chlorogenic, neochlorogenic and 4-caffeoylquinic acid) were also identified [57].

These metabolite classes have not yet been referenced for other edible *Cornus*. However, it can be noted that among these common metabolites, only ursolic, malic, citric and neochlorogenic acid have been identified in *C. mas*, *C. officinalis* and *C. kousa*. Table 5 reports the main acids and phenolic acids identified in *Cornus* fruits.

### 4.5. Carotenoids

Carotenoids were identified only in two species of *Cornus* edible fruits, *C. mas* and *C. kousa*. Ten carotenoids, namely *β*-carotene, *β*-carotene-5,6-monoxide, *β*-cryptoxanthin, lutein, lutein-5,6-epoxide, (9*Z*, 9′*Z*)-lutein, (13*Z*, 13′*Z*)-lutein, (all-*E*)-neoxanthin, (9′*Z*)-neoxanthin, luteoxanthin, are reported in the hydroalcoholic extract of *C. mas* fruits treated with hexane [93]. Six carotenoids, namely lutein, neoxanthin, violaxanthin, antheraxanthin, zeaxanthin, *α*-carotene, and *β*-carotene, are found in *C. kousa* fruits [57].

### 4.6. Iridoids

The taxonomic classification of the *Cornus* genus belongs to the super division Spermatophyta, division Magnoliophyta, class Magnoliopsida (dicotyledon), sub-class Rosidae, order Cornales, family Cornaceae and genus *Cornus* L. [1]. This classification is in accordance with iridoids as taxonomic markers of this genus [94,95]. Since Jensen et al. [94] and Bate-Smith et al. [95], it has been established that the taxonomically informative chemical compounds reported are cornin and loganin. Morroniside, whose formation was established in vivo derivation from secologanin, is only found in *C. officinalis* [94].

Cornel iridoid glycoside is one of the main components of *C. officinalis,* together with morroniside and loganin [96].

To the best of our knowledge, among the edible *Cornus*, the presence of iridoids in the fruits has only been investigated in the species *C. mas* and *C. officinalis*.

Table 6 shows that all identified iridoids have a C10 skeleton. In the fruits of *C. mas*, the taxonomic markers of the genus *Cornus* were identified, as well as some derivatives such as sweroside, loganic acid and cornuside, an iridoid frequently found in the genus *Cornus*. Loganic acid, loganin, and sweroside have been detected in the hydroalcoholic extract of fruits and juice [52,82].

In addition to the iridoids found in *C. mas* extract, many other iridoids are described in the fruits of *C. officinalis*. For example, derivatives of loganin esterified with malic acid are present [105]. Secoiridoids are numerous; in addition to those mentioned above, demethoxy-cornuside, secologanoside, secoxyloganin were identified. However, the main secoiridoid is morroniside, whose isomeric forms are in equilibrium (*α* and *β* morroniside) [105]. Probably due to the solvent’s nature, the *α* and *β* isomeric forms of morroniside ethyl and methyl were also isolated.

*α*,*β*-Morronisides are precursors of many derivatives. Glycosylation reactions between the C-7 of morronisides and glucose or fructose alcohol functions yielded twelve morroniside diglycosides (Cornusdiglycosides A−J) [114]. The alcohols of the morronisides reacted with the C-7 carbon of a second morroniside, forming dimers of methyl and ethyl morronisides (Cornuside A-J, Williamsoside D). The links between the two morronisides units are (6′-*O*-7″) and (2′-*O*-7″) [104]. Similarly, iridoid dimers of the cornuside-morroniside (Cornusdiridoid A−F) [99] and loganin-morroniside (Cornuside L-O) [51,104,105] types were also formed. The links are of the type (2′-*O*-7′′′), (3′-*O*-7′′′), (4′-*O*-7′′′), (6′-*O*-7′′′) and (6′-*O*-7′′′), (2′-*O*-7″), (4′-*O*-7″), respectively. Moreover, the tryptamine also reacted with morroniside, forming monoterpene indol alkaloids (MIA) in the form of 3*β*(R)-vincosamide, 7-epi-javaniside and javaniside [99]. In addition, unusual compounds such as logmalicides A and B [8], cornusfurosides A−D [110], and cornus phenosides A-I [111] were identified.

### 4.7. Other Constituents

Glucose, fructose and sucrose are present in high concentrations in the hydroalcoholic extract of *C. mas* fruits. Glucose is the major carbohydrate, followed by fructose and sucrose [74,115]. Contrary to what is observed in *C. mas*, fructose is the main sugar in *C. kousa*, followed by glucose. *C. kousa* presents a sweet taste and a higher total sugar content than other *Cornus* edible fruits [57]. In *C. officinalis* fruits, only sucrose is found [64]. *C. mas* fruits are rich in potassium, calcium, magnesium and sodium content. Other minerals such as phosphorus, iron, zinc, copper and manganese are present in smaller quantities. Variability was found in dependence on the collection site [74,116,117,118]. For example, fruits from the Czech Republic showed a variability in potassium (3798–3411 mg/kg), calcium (656–301 mg/kg), magnesium (290–241 mg/kg), and sodium (82–58 mg/kg) depending on the type of cultivar [116]. Fruits from cultivars of Serbia exhibited 5609–1845 mg/kg of potassium, 466–27 mg/kg of calcium, 315–40 mg/kg of sodium, and 161–10 mg/kg of magnesium [74]. Variability in mineral content was also found in the samples collected in Croatia and Greece. In the *C. mas* fruits collected in Croatia, the main mineral was potassium (4019 mg/kg), followed by calcium (2074 mg/kg), magnesium (288 mg/kg) and sodium (22.9 mg/kg) [118]. A different composition was reported for fruits from Greece, with potassium (1320–880 mg/kg), phosphorus (90–80 mg/kg), magnesium (50–40 mg/kg), iron (45–19 mg/kg) and calcium (30–20 mg/kg) identified as the main minerals [117]. Compared with other fruit juices (pear, plum and apple juices), the *C. mas* fruit juice was richer in various minerals such as potassium, calcium, sodium, iron, zinc, copper and manganese [119]. There was little difference in the mineral composition between *C. mas* and *C. officinalis* fruits. These last species did not demonstrate the presence of sodium and phosphorus in their composition [120].

The vitamins identified in the aqueous extracts of *C. mas* fruits are ascorbic acid, *α*-tocopherol, biotin, and riboflavin. Their concentration varies depending on the cultivar and climatic conditions [121]. As far as *C. officinalis*, only γ-tocopherol has been identified in the essential oil obtained from its fruits, and other data concerning vitamins are completely missed [122]. Regarding *C. kousa*, ascorbic acid, *α*-tocopherol and γ-tocopherol are found in its fruits [57]. In a recent study, only ascorbic acid was described in *C. sanguinea* [71].

Most published works do not mention the quantification of isolated metabolites or the different compounds in the extracts. The study of *C. mas* cultivars showed that the concentration of the latter changes, and these differences might explain the role of metabolites. For example, for fruits from *C. mas* cultivars, it has been shown that total phenolic content and ascorbic acid content are correlated with high biological efficiency in antioxidant activity [3].

## 5. Biological Properties

*C. mas* demonstrated antioxidant, antibacterial, antidiabetic, hypolipidemic, anti-inflammatory, anticancer, and anticoagulant properties [1]. Additionally, *C. officinalis* is known to have different biological properties, such as antimicrobial, antineoplastic, hypoglycaemic, antioxidant, anti-inflammatory, cardioprotective, and neuroprotective activities, and has been traditionally used to improve and protect kidney and liver functions [48]. Conversely, few studies assessed the bioactivities of *C. sanguinea*, *C. kousa* and *C. controversa* [123] until now. Herein, we described the most investigated properties. The main investigated biological activities are summarized in Table 7.

### 5.1. Antioxidant Activity

The fruits of *C. mas* are a good source of antioxidants [3,70,81,124,125,126,127]. Bayram and Ozturkcan [75] reviewed the antioxidant properties of different extracts, juice, liqueur and wine of *C. mas*. In a study by Serteser et al. [127], the hydroalcoholic extract of fruits from *C. mas* collected in Turkey showed an interesting inhibition against DPPH radical and H_2_O_2_ activity and a high Fe^2+^ chelating activity.

Another study analyzed the antioxidant capacities of the Cornus species typically found in the Mediterranean area. The hydroalcoholic extract obtained by *C. mas* fruits from Northern Greece showed high protection (98.6%) in the deoxyribose protein assay, along with the Hull thornless blackberry (red-black, *Rubus fruticosus*) (98.9%).

Raspberries and gooseberries presented a low capacity [125]. The antioxidant activity is influenced by the phytochemical content, which is influenced by different factors, including genotype, site of collection, maturity stage, climatic conditions, and extraction procedures. The methanol extract of *C. mas* air-dried fruits showed a lower FRAP value (83.9 μM ascorbic acid equivalent (AAE)/g, dry weight (DW)) compared with lyophilized fruits (FRAP value of 190 μM AAE/g, DW) [81,125].

During the drying process, a decomposition of bioactive compounds such as anthocyanins and ascorbic acid was observed, which is the reason for a reduced FRAP activity. Tural and Koca [70] investigated various *C. mas* genotypes of Turkey and reported different FRAP activity (16.21–94.43 μM AAE/g, fresh weight (FW)). In addition, the presence of pelargonidin 3-*O*-glucoside was the main pigment found in these fruits. Stanković et al. [128] used solvents of different polarities, such as methanol, water, ethyl acetate, petroleum ether, and acetone, to obtain different *C. mas* fruit extracts. The highest antioxidant activity was reported for the ethyl acetate extract with an IC_50_ value of 11.06 μg/mL in the DPPH test.

This activity could be associated with total phenolic content (179.05 mg gallic acid equivalent/g) and flavonoid concentration (41.49 mg rutin equivalent/g). Moreover, the DPPH radical scavenging activity of the ethyl acetate extract is similar to standard compounds (butylated hydroxyanisole (BHA; 5.39 μg/mL), rutin (9.28 μg/mL) and chlorogenic acid (11.65 μg/mL).

Serteser et al. [127] compared the antioxidant activity of the hydroalcoholic extract of *C. sanguinea* fruits with other plants collected in Turkey, demonstrating that *C. sanguinea* has the highest DPPH radical scavenging activity. A similar trend was observed with Fe^2+^ chelating and H_2_O_2_ inhibition activities. Moreover, this extract was more active in the H_2_O_2_ inhibition test than extracts of other plants. The methanol extract of *C. sanguinea* fruits collected in Iran showed an interesting antioxidant activity in the DPPH test, with an IC_50_ value of 94.83 μg/mL [123]. A lower DPPH radical scavenging activity was found by Stanković and Topuzović [129], who analysed different *C. sanguinea* fruit extracts. The acetone extract showed a high capacity to reduce DPPH radicals with an IC_50_ value of 247.83 μg/mL, followed by methanol extract (358.59 μg/mL), water extract (384.85 μg/mL), ethyl acetate extract (537.83 μg/mL) and petroleum ether extract (1202.85 μg/mL).

Previously, Lee et al. [130] studied the antioxidant activities of n-hexane, chloroform, ethyl acetate, ethanol, and water extracts of *C. officinalis* dried fruits. Antioxidant activity was investigated in a linoleic acid emulsion system by measuring the peroxide value, while radical scavenging activity was determined through the DPPH test. All extracts reported significantly higher DPPH radical scavenging activity than the positive control.

Water and ethanol extracts exhibited higher activities compared to other extracts. The same trend was observed in the linoleic acid emulsion system [130]. Additionally, in the same study, the protective effects of *C. officinalis* extract against apoptotic damage in H_2_O_2_-treated human umbilical vein endothelial cells (HUVECs) were assessed. Ethanol extract decreased cell death, preserving morphological normality by eliminating hydroxyl radicals.

In another study, Kim et al. [131] investigated the in vivo antioxidant effect of *C. officinalis* aqueous extract. In diabetic rats treated with a dose of 500 mg/kg, the activities of xanthine oxidase (XO), catalase (CAT), and glutathione-*S*-transferase (GST) were lower than the diabetic group. In contrast, the superoxide dismutase (SOD) activity increased by 26%. Thus, it is possible to deduce that antioxidant activity could be linked to the regulation of enzymes.

Successively, West et al. [102], investigated the antioxidant capacities of *C. officinalis* juice. This juice showed a promising DPPH radical scavenging activity (82.32 percent DPPH radical scavenging activity), moderate reducing power (30.71 mmol Fe^2+^/mL) and oxygen radical absorbance capacity (22.31 μmole Trolox equivalent/mL). Interestingly, the *C. officinalis* juice reducing power activity is greater than reported for other juices such as apple, pomegranate, açaí, black cherry, blueberry, cranberry, Concord grape, and orange juices [132]. The hydroalcoholic (70% ethanol) extract of *C. officinalis* fruits showed an interesting antioxidant activity in the DPPH test, with an IC_50_ value of 99.32 μg/mL. In addition, in the ABTS test, this extract demonstrated a scavenging activity of 40.7% at a 100 μg/mL concentration. Additionally, it exhibited a high ability to reduce ferric complex (241.5 mM), similar to the positive control (256.1 mM). This high antioxidant activity should be associated with phenolic compounds found in this species [133].

### 5.2. Antidiabetic and Anti-Obesity Activities

Diabetes mellitus is a chronic metabolic disorder of multiple aetiology. Type 2 diabetes represents over 90% of diabetes and is associated with reduced sensitivity to insulin by peripheral tissues. One therapeutic approach for treating type 2 diabetes is to reduce postprandial hyperglycemia by inhibiting carbohydrate-hydrolysing enzymes, such as *α*-glucosidase and *α*-amylase [134].

*C. officinalis*, whose dried and mature fruits have traditionally been used for preventing diabetes mellitus, diabetic nephropathy, chronic inflammatory diseases and liver disease, is mainly distributed in Eastern Asia, Southern and Central Europe, and Eastern North America [135]. The antidiabetic properties of *C. officinalis* were studied in non-insulin-dependent diabetes mellitus (NIDDM) rats by oral administration (1 once daily for 30 d). In NIDDM, the expression of GLUT4 mRNA was significantly reduced. Still, the administration of *C. officinalis* alcohol extract notably increased the GLUT4 mRNA and protein expression, thus increasing insulin secretion and accelerating the metabolism of glucose [136].

Treatment with *C. officinalis* extract (oral administration of 300 mg/kg) in streptozotocin (STZ)-induced diabetic rats for 4 weeks reduced blood glucose and triglycerides (TG), significantly alleviating polyuria, polyphagia, polydipsia and weight loss. In addition, the oral administration of *C. officinalis* protected against damage to diabetes-induced pancreas and kidney injury, increased the pancreas’s beta cells, and consequently increased insulin levels [137]. Previously, Yamabe et al. [138] assessed the antidiabetic properties of *C. officinalis* fruits in STZ-induced diabetic rats by gavage. The experimental groups that received the extract at doses of 50, 100, and 200 mg/kg body for 10 days presented a reduction in proteinuria, hyperglycemia, renal advanced glycation end-product (AGE) formation, and the expression of related proteins, such as the receptor for AGEs, nuclear factor-κB (NF-κB), transforming growth factor-*β*1, and Nε-(carboxymethyl)lysine [138].

Moreover, Park et al. [139] demonstrated that *C. officinalis* extract could reduce serum glucose levels by inhibiting the *α*-glucosidase enzyme. In another study, the insulin-mimetic action of *C. officinalis* on dexamethasone and 8-bromo-cAMP-induced phosphoenolpyruvate carboxykinase (PEPCK) expression in H4IIE cells (rat hepatoma cell) was studied. *C. officinalis* methanol extract showed potent insulin mimic activity on PEPCK expression, increasing the cell viability and promoting insulin secretion [140]. The therapeutic effects of *C. officinalis* extract were also confirmed by Gao et al. [141]. Extract was administered at doses of 100, 200, or 400 mg/kg by oral gavage to STZ-induced diabetic rats. Reduction in blood glucose, high-density lipoprotein (HDL), low-density lipoprotein (LDL), TG, urinary protein levels and water consumption was observed. At the highest doses tested (200 and 400 mg/kg), creatinine and serum albumin levels were significantly reduced. The expression of peroxisome proliferator-activated receptor-gamma (PPARγ) was improved with SOD, CAT and glutathione peroxidase (GPx) activities. Thus, decreasing oxidative stress and enhancing the PPARγ expression can improve diabetic nephropathy.

Iridoids and secoiridoids are characteristic and active constituents of *C. officinalis*. They have been reported to possess anti-diabetic, antioxidant and anti-inflammatory properties [114]. The iridoid glycosides cornusdiglycosides A−J, isolated from the fruits of *C. officinalis*, were recently assessed for their potential *α*-glucosidase inhibitory activity. All iridoids inhibited *α*-glucosidase, with IC_50_ values ranging from 78.9 to 162.2 μM [114]. Cornusdiglycosides A−H revealed better inhibitory activity than acarbose, used as a positive control.

Successively, six unusual cornuside-morroniside secoiridoid dimers, namely cornusdiridoid A−F, isolated from the fruits of *C. officinalis*, were investigated as potential a-glucosidase inhibitory agents [99]. Among the tested compounds, cornusdiridoid A−C exhibited the most promising activity with IC_50_ values of 243.5–267.1 μM (acarbose, 276.3 μM).

An et al. [142] obtained total saponins from *C. officinalis* by using ultrasonic microwave-assisted extraction. This extract, purified by a non-polar copolymer styrene type macroporous resin (HPD-300), improved the glucose and lipid metabolism of streptozotocin-induced diabetic mice and ameliorated liver and pancreas damage, inflammation as well as oxidative stress. The *C. officinalis* total saponin extract regulated insulin receptor-, phosphatidylinositol 3-kinase-, glucose transporter 4-, and protein kinase B-associated signaling pathways.

A literature survey also reported several studies that analysed *C. mas* for its potential antidiabetic activity. The hydroalcoholic extract of *C. mas* fruits (100 mg/kg, intraperitoneal (i.p.)) exhibited antidiabetic activity in alloxan-induced diabetic rats by reducubg serum glucose, LDL, TG, and very-low-density lipoprotein (VLDL) levels and by increasing HDL [143].

The oral administration of *C. mas* fruits (5, 10 and 15 g/meal) as a diet supplement for 20 days in hamsters decreased body weight and increased insulin levels [144]. Successively, Shamsi et al. [145] demonstrated that the oral administration of *C. mas* fruits (2 g/kg/day (d) for 4 weeks in alloxan-induced diabetic rats exhibited antidiabetic effects comparable to the positive control glibenclamide (0.6 mg/kg/d) [146].

In a clinical study, 60 patients with type 2 diabetes were randomly assigned to two groups receiving a *C. mas* anthocyanin-rich extract or placebo capsules (2 capsules twice daily after main food) orally along with their usual diet and physical activity. Each capsule contained 150 mg of anthocyanins isolated from the aqueous ethanol extract of *C. mas* fresh fruits. After 6 weeks, a significant increase in insulin levels and a decrease in haemoglobin AIc (HgbAIC) and TG levels were observed in the treated group in comparison to the placebo. These results suggested that the consumption of *C. mas* fruits might improve glucose intolerance in type 2 diabetic patients by increasing insulin levels and reducing the HgbAIC and TG levels [147].

Anthocyanins, of which *C. mas* fruits are very rich, have been shown to reduce the development of obesity through suppressed fat accumulation, significantly increasing the enzyme activity responsible for lipolysis of TG [148,149]. Jayaprakasam et al. [150] demonstrated that cyanidin and delphinidin glucosides stimulated insulin production in vitro. In contrast, Zhang et al. [151] showed that ursolic acid decreases blood glucose through insulin receptor phosphorylation and the stimulation of glucose uptake.

The consumption of lyophilized *C. mas* fruit powder (doses of 500 and 1000 mg/kg bw/d) reduced mice’s fasting plasma glucose levels after 12 weeks without affecting the homeostatic model assessment of insulin resistance [152].

Similar results were obtained in the study by Dzydzan et al. [153], which demonstrated that red and yellow C. mas fruit ethanol extracts (20 mg/kg intraperitoneal injection (i.p.) for 14 days) were able to reduce the fasting plasma glucose levels and increase glucose intolerance. Moreover, the hydroalcoholic (75% ethanol) extract of *C. mas* fruits (100 mg/kg bw/d for 3 days) decreased serum glucose, TG, LDL, and VLDL levels and HDL levels. These effects were similar to glibenclamide treatment (50 mcg/kg i.p.) (an anti-diabetic drug), while reductions in TG and VLDL by hydroalcoholic extract of *C. mas* fruits were greater than glibenclamide [143].

Different extracts (obtained by different extraction procedures) and constituents of *C. mas,* including anthocyanins, iridoids, and ursolic acid, showed anti-diabetic effects by using several in vitro and in vivo procedures. However, more studies are needed to confirm which extract or compound is more effective.

### 5.3. Hypolipidemic and Anti-Atherosclerotic Properties

The effects of *C. mas* fruits on hypercholesterolemic rabbits were evaluated [154]. The administration of *C. mas* fruits to rabbits (1 g/kg body weight (b.w.)/d for 60 days) decreased serum fibrinogen levels. This effect was better than the anti-fibrinogenic drug, lovastatin (10 mg/kg b.w./d for 60 days). Therefore, the consumption of *C. mas* fruits as a diet supplement might be beneficial to reduce the risk of cardiovascular diseases in atherosclerotic patients. Authors applied the same protocol to rabbits, with a significant decrease in LDL, malondialdehyde (MDA), TG, total cholesterol (TC), fibrinogen and atherogenic index parameter. Additionally, an increase in antioxidant activity was observed. Probably, this effect is related to the number of phenolic compounds and vitamins (ascorbic acid and tocopherol) [155].

In another study on postmenopausal women, participants were randomly separated into two groups. One group received *C. mas* fruit extract (300 mg) 3 times a day for 8 weeks, and another group received a placebo. After the treatment, body weight, waist circumference, BMI, LDL/HDL *ratio*, TC/HDL *ratio*, and fibrinogen decreased. Moreover, *C. mas* supplementation increased Apo A1 and HDL levels compared to the control group. However, although both groups’ fasting plasma glucose levels decreased, no change in leptin levels, serum insulin, insulin resistance index and insulin sensitivity were appreciated. This work revealed that daily consumption of *C. mas* fruits might help decrease blood sugar parameters and lower the risk of cardiovascular diseases. Successively, Sozanski et al. [68] investigated the protective effect of *C. mas* fruits against atherosclerosis and hypertriglyceridemia in a rabbit model. Fruits (100 mg/kg b.w.) or simvastatin (5 mg/kg b.w.) were administered orally for 60 days. A significant increase in liver peroxisome proliferator-activated receptor α (PPARα) protein expression and a significant decrease in serum triglyceride levels (44%), pro-inflammatory cytokines, interleukin-6 (IL-6), and tumor necrosis factor-α (TNF-α) was demonstrated. Based on these results, *C. mas* fruits showed protective effects against diet-induced hypertriglyceridemia and atherosclerosis across increased PPARα protein expression by regulating oxidative stress and inflammation [68]. It was demonstrated that the activation of PPARα leads to the increased tissue-specific expression of crucial genes involved in fatty acid uptake and *β*-oxidation [156].

PPARα decreases triglycerides by increasing free fatty acid *β*-oxidation, hepatic lipoprotein lipase expression, and expression of apolipoprotein V, and by decreasing expression of apolipoprotein CIII. Furthermore, PPARα activation regulates HDL metabolism by stimulating hepatic expression of apoA-I and apoA-II, thus raising HDL production in the liver, promoting HDL-mediated cholesterol efflux from macrophages, and inhibiting cellular cholesteryl ester formation activity, as well as contributing to an enhanced efflux of free cholesterol to extracellular receptors. PPARα activation may indirectly influence atherosclerosis development through the effects of glucose and lipid homeostasis on adipose tissue, liver, and skeletal muscle. In addition, PPARα activation may directly affect inflammation by modification of NF-κB and activator protein-1 (AP-1).

By regulating oxidative stress and inflammation, it is possible to conclude that both loganin and cornuside are responsible for the hypolipidemic activity of the extract. In fact, loganin showed anti-inflammatory capacities in various disease models, including acute pancreatitis and Parkinson’s disease ([157,158]. The iridoid was able to inhibit inflammatory cytokines and deactivation of the NF-κB signalling pathway. Moreover, it was demonstrated that the administration of loganin could improve memory-enhancing long-term memory in hippocampal tissues [159].

Cornuside was demonstrated to dilate vascular smooth muscle through endothelium-dependent nitric oxide signalling [160] and showed anti-inflammatory activity in the lipopolysaccharide (LPS)-induced inflammation model via the inhibition of NF-κB activity [161]. Previously, Kang et al. [162] observed that the addition of cornuside to human umbilical vein endothelial cell cultures reduced the mRNA expression of vascular cell adhesion molecule-1 (VCAM-1) and intercellular adhesion molecule-1 (ICAM-1) and diminished TNFα-induced NF-κB activation. In a carrageenan-induced mouse paw edema model, loganic acid exhibited a strong anti-inflammatory activity [163].

Loganic acid and cornuside were shown to increase PPAR-α levels by reducing atherosclerotic plaque formation in cardiovascular diseases [164].

The hypocortisol and hypolipidemic effects of *C. mas* fruits (5–15 g/daily) were studied in a hamster model [165]. At the highest dose, a decrease in cholesterol (108.3 mg/mL) and LDL (21 mg/mL) level were observed, as well as increased HDL (54 mg/mL) and TG (191.7 mg/mL) levels. The hamsters treated with *C. mas* fruits also showed a decrease in cortisol levels (28.8 ng/mL) compared with control (45.8 ng/mL).

As demonstrated by the different described works, the consumption of *C. mas* fruits may benefit lipid disorders. A recent systematic review and meta-analysis of the effects of cornelian fruit supplementation on the blood lipid profile of animal studies revealed that the supplementation considerably reduced the levels of LDL, TG, and cholesterol and increased HDL levels compared to the control group [166].

Consequently, these findings suggest that *C. mas* fruits supplementation may have lipid-modifying properties, which might be because these fruits are a good source of polyphenols and anthocyanins. However, long-term clinical studies and well-designed human studies should be necessary to further evaluate the impacts of cornelian consumption on various health outcomes.

*C. officinalis* was evaluated for its hypolipidemic activity [167]. The extract was able to reduce serum and hepatic total cholesterol levels and lipid peroxidation at an oral dose of 50, 100, or 200 mg/kg for 10 days. Moreover, expression of the sterol regulatory element-binding protein (SREBP)-2 was reduced, whereas expression of the PPARα protein was enhanced. These results suggest using *C. officinalis* to prevent cardiovascular disease by controlling cholesterol levels and the expression of proteins related to lipid metabolism.

### 5.4. Neuroprotective Effects

A literature survey revealed the presence of several studies that have proposed *C. officinalis* and *C. mas* as potential therapeutic agents to treat neurodegenerative diseases, including Alzheimer’s disease (AD).

By using APP/PS1/tau triple transgenic (3 × Tg) mice as a model of AD, Yang et al. [168] revealed that cornel iridoid glycoside, the dominant active constituent of *C. officinalis*, could ameliorate learning and memory impairments of 3 × Tg mice by down-regulating the expression of Aβ and full-length amyloid precursor protein, as well as decreasing the hyper-phosphorylation of tau protein. These results were confirmed by Ma et al. [169], which demonstrated that this iridoid could reduce the hyperphosphorylation and aggregation of tau protein in the amygdala, prevent neuronal loss, and suppress the hyperactivity phenotype of AD. Tian et al. [170] used immobilization stress that induced depressive-like and anxiety-like behaviours and hippocampal neuronal damage in rodents to investigate the protective properties of *C. officinalis* extract on nerve injury. Results showed that *C. officinalis* successfully alleviated oxidative stress, decreased immobility time in the forced swim test, increased serotonin levels and significantly reduced corticosterone and *β*-endorphin levels. Iridoid glycosides of *C. officinalis* could reduce corticosterone-induced PC12 cell damage [97]. Corticosterone-induced PC12 injury is considered an in vitro experimental model for depression, and PC12 cells have typical neuron characteristics and are widely used to study neurological diseases [171]. In another study [172], a decoction of *C. officinalis* fruits exhibited protective effects against neurodegenerative diseases. Treatment with an extract (60 µg/mL) stimulated neurite extension similarly to a prominent neurotrophic factor, increasing the percentage of adrenal gland pheochromocytoma (PC12) cells bearing neurites.

The administration of freeze-dried *C. mas* fruits protected the brain tissue by reducing the amount of free radicals generated in the brain tissue. These effects were demonstrated in three types of diet: control, fructose and high-fat diets in rats with increased activity of catalase (CAT) and paraoxonase (PON) in brain tissue and decreased levels of the protein carbonyl group and thiol groups in brain tissue as well as in plasma [173]. PON is responsible for the anti-atherogenic effects of HDL in blood, possesses anti-inflammatory properties and prevents the oxidation of lipoproteins [174]. Phenolic compounds and ascorbic acid in *C. mas* fruits probably play a significant role in increasing PON-1 levels [175].

Some *Cornus* constituents have been studied as potential neuroprotective agents. Among them, morroniside can promote both the proliferation and differentiation of endogenous neural stem cells and the migration of neural stem cells in vivo. Nerve growth factor (NGF) and brain-derived neurotrophic factor (BDNF) are important bioactive molecules in the nervous system for their role in developing the central nervous system while maintaining the normal functions of the mature central and peripheral nervous system neurons. Morroniside enhanced the function of NGF and the expression of BNDF [176,177]. The effects of morroniside on the expression of the Wnt7a gene and adenomatous polyposis coli (APC) were assessed in rats with focal cerebral ischemia-reperfusion injury [178]. The Wnt is a pathway that can regulate cell proliferation and differentiation during the growth and development of humans and animals, particularly in brain plasticity. Wnt7a is an important ligand that can regulate the acceleration of cell growth and differentiation and inhibit glial regeneration. Conversely, the APC gene inhibits the Wnt signaling pathway. Morroniside promoted the expression of Wnt7a and inhibited the expression of APC.

### 5.5. Antimicrobial Activity

The *n*-hexane extract of *C. mas* fruits showed significant antibacterial activity against Gram-positive *Staphylococcus aureus* and Gram-negative *Escherichia coli* at the tested dose of 10 μL/disc [179]. Methanol and ethanol extracts of *C. mas* fruits revealed antibacterial activity against *E. coli*, *S. aureus*, and *Pseudomonas aeruginosa*, but demonstrated no antifungal properties against *Candida albicans* and Aspergillus fumigates. However, *Streptococcus pyogenes* and *Trichophyton mentagrophytes* resulted in resistance [180].

The antimicrobial effects of a hydroalcoholic extract of *C. mas* fruits were successively investigated against 13 species of bacteria and yeast [49]. The Gram-positive strains *Listeria monocytogenes*, *Bacillus cereus*, *S. aureus*, and *Sarcina lutea* and the Gram-negative strains *Proteus vulgaris*, *Shigella sonnei*, and *Salmonella enteritidis* were the most sensitive. A positive correlation between antimicrobial activity and total phenol content was found. The scientifically proven antibacterial activity justified using *C. mas* fruits in traditional medicine to treat gastrointestinal disorders and diarrhoea, skin diseases, and urinary infections [181,182,183,184]. The antimicrobial effect of apple juice supplemented with *C. officinalis* fruits against *E. coli* O157:H7 was evaluated [185]. Results showed that adding *C. officinalis* fruits inactivates *E. coli* O157:H7 in apple juice. The most promising results were observed at 21 °C, while at 7 °C, only a reduction of 2.3 log cfu/mL was observed. Therefore, *C. officinalis* fruit extract can be used with temperature and storage time conditions to inactivate *E. coli* O157:H7 in apple juice.

### 5.6. Other Bioactivities

The hydroalcoholic extract of *C. mas* fruits (5 μg/mL) exhibited cytotoxic activity against breast adenocarcinoma (MCF-7), prostate adenocarcinoma (PC-3), ovarian cancer (SKOV-3), and non-small cell lung cancer (A549) cells [186]. The mean growth inhibition was 81.8, 81.9, 81.6, and 79.3% in the SKOV3, MCF-7, PC-3, and A549 cell lines, respectively. Previously, Chang et al. [187] demonstrated that a decoction of *C. officinalis* fruits at the dose of 100 µg/mL was able to inhibit hepatocellular carcinoma cell (58.1%) and leukemic cell (51.2%) growth.

The hepato-protective effect of *C. mas* was also proven. Fruits were administered at 200 and 500 mg/kg b.w., ameliorating the levels of aspartate transaminase (AST), alanine transaminase (ALT) and alkaline phosphatase (ALP) and reducing the oxidative stress and elevated hepatic MDA content in CCl4-induced hepatotoxicity in rats by Alavian et al. [188]. Another research group demonstrated that the hydroalcoholic extract of *C. mas* fruits exerted renal-protective effects by improving renal lesions, antioxidant enzymes, lipid peroxidation-induced MDA levels, and other biochemical parameters, including creatinine, uric acid and serum urea levels [189].

The oral administration of a hydroalcoholic extract of *C. mas* fruits (50, 200 and 400 mg/kg b.w./d) exhibited a significant reduction in the platelet distribution width (PDW) [190], a specific marker of platelet activation [191]. This marker is activated when blood coagulation is necessary and consequently decreases with platelets’ inactivation.

In rats, CCl_4_ injection can decrease antioxidant enzymes and increase creatinine kinase, lactate dehydrogenase and MDA, with consequent damage to different tissues, including the heart. Treatments with the hydroalcoholic extract of *C. mas* fruits at a dose of 300 and 700 mg/kg b.w./d for 16 days in CCl4-induced intoxicated rats showed cardioprotective effects. These effects were linked to attenuating myocardial lipid peroxidation levels and recovering the enzymatic defense system by increasing the levels of SOD, CAT, and GPx and modulating cardiac tissue’s bioenergetics state [192].

Loganin, one of the most common iridoid glycosides isolated from *Cornus* species, was recently investigated on angiotensin II-induced cardiac hypertrophy [193]. Angiotensin II is a significant component of the renin-angiotensin system and plays an important role in triggering hypertension and myocardial hypertrophy. Loganin was demonstrated to inhibit angiotensin II–provoked cardiac hypertrophy and cardiac damage in the H9C2 cell line in mice. It showed cardio-protective effects through a decrease in pro-inflammatory cytokine secretion, the suppression of the phosphorylation of critical proteins including STAT3, JAK2, IκBα, and p65, and the attenuation of cardiac fibrosis. These promising results are supported by previous works that have shown the ability of loganin to suppress inflammatory responses by downregulating the activation of JAK2/STAT3 and NF-κB signaling pathways and decreasing the production of inflammatory cytokines [194,195,196,197].

The administration of loganic acid, isolated from *C. mas* fruits, at a dose of 0.7% aqueous solution containing 0.15% sodium hyaluronate significantly reduced (25%) conjunctivitis-induced intraocular pressure (IOP) of the eye in a rabbit model [82].

This activity is of certain interest as a possible treatment for diabetic retinopathy. The decrease in IOP was probably ascribed to reducing nitric oxide [198,199]. A similar effect was also observed in a glaucoma model. Glaucoma is characterised by high levels of nitric oxide that decrease after loganic acid treatment [198,199].

Osteoporosis is a chronic illness closely linked with the aging processes. Based on traditional medicine and modern findings in the pathogenesis and treatment of osteoporosis, a variety of natural products present in preparations of this plant play a beneficial role.

*C. officinalis* significantly inhibits receptor activator of nuclear factor-κB ligand (RANKL)-mediated osteoclast differentiation in a dose-dependent manner and RANKL-induced phosphorylation of p38 and c-JUN N-terminal kinase. In addition, the protein expression of c-Fos and nuclear factor of activated T cells cytoplasmic 1 (NFATc1) is suppressed due to the RANKL-induced degradation of I-κB kinase [200]. The effects of the main constituents of *C. officinalis* fruits, such as morroniside, on osteoarthritis in human chondrocytes were studied. Morroniside supported the survival of chondrocytes with articular cartilage thickness, increasing the levels of type II collagen and improving the level of proteoglycans in the cartilage matrix [201].

Studies conducted on *C. officinalis* revealed a reduction in the incidence of infections or allergic illnesses by stimulating the innate immune system. In particular, Kim, Kim, and Lee [202] evaluated the therapeutic effects of *C. officinalis* in a mouse model of allergic asthma. This model demonstrated reduced production of Th2 cytokines (IL-5), eotaxin, and specific immunoglobulin E (IgE) and reduced eosinophil infiltration. *C. officinalis* fruits improve non-specific immunity, specific humoral immunity, and cellular immunity, as confirmed in a previous study conducted by Du et al. [203] in immuno-depressed mice.

**Table 7:** The main biological activities described for *Cornus* species.

	Extracts or Compounds	Country	Effects	Reference
***C. mas***				
**Antioxidant activity**				
	Hydroalcoholic extract of dried fruits	Turkey	Antioxidant activity (DPPH assay: IC_50_ of 1.078 mg/mL) and high inhibition against H_2_O_2_ activity (74.35%)	[127]
	Hydroalcoholic extract of dried fruits	Greece	Antioxidant activity in FRAP test (83.9 μM AAE/g of DW) and deoxyribose test (98.6%)	[125]
	Methanol extract of fresh fruits	Iran	Antioxidant activity in FRAP assay (190 μM AAE/g of DW) and DPPH assay (3.95–9.67 mg/mL)	[81]
	Methanol extract of fresh fruits	Turkey	Antioxidant activity tested with FRAP test (16.21–94.43 μM AAE/g of FW) and DPPH test (IC_50_ value of 0.29–0.69 mg/mL)	[70]
	Methanol, aqueous, ethyl acetate, petroleum ether and acetone extracts of fresh fruits	Serbia	Antioxidant activity in DPPH assay (IC_50_ of 251,86, 518.47, 11.06, 107.99, and 285.98 μg/mL for methanol, aqueous, ethyl acetate, acetone, petroleum ether extracts, respectively)	[128]
**Antidiabetic and anti-obesity activities**				
	Hydroalcoholic extract of fresh fruits		Antidiabetic activity in alloxan-induced diabetic rats by reductions in serum glucose, LDL, TG, and VLDL levels and increase in HDL	[143]
	Fresh fruits	Iran	Decrease in body weight and increase in insulin levels	[144,145,146]
	Hydroalcoholic extract of fresh fruit	Iran	Increase in insulin level, decrease in HgbAIC and TG levels	[147]
	Aqueous extract of fresh fruits	Slovakia	Reduction in plasma glucose levels	[152]
	Ethanol extract		Reduction in plasma glucose levels and increase in glucose intolerance	[153]
	Cyanidin and delphinidin glucosides		Stimulation of insulin production	[150]
	Ursolic acid		Decrease in blood glucose and stimulation of glucose uptake	[151]
**Hypolipidemic and anti-atherosclerotic properties**				
	Dried fruits	Iran	Decrease in serum fibrinogen levels, LDL, MDA, TG, TC, fibrinogen and atherogenic index parameter	[154,155]
	Fruit extract	Iran	Decrease in body weight, waist circumference, BMI, LDL/HDL ratio, TC/HDL ratio, and fibrinogen, and increase in Apo A1 and HDL levels	[204]
	Fresh fruits	Poland	Protective effects against diet-induced hypertriglyceridemia and atherosclerosis through an increase in PPARα protein expression and a significant decrease in serum triglyceride levels, pro-inflammatory cytokines, IL-6, and TNF-α	[68]
	Fresh fruits	Iran	Decrease in cholesterol, LDL, and cortisol levels; increase in HDL and TG levels	[165]
	Loganin		Inhibition of inflammatory cytokines and deactivation of NF-κB signalling pathway	[157,158]
	Cornuside		Dilated vascular smooth muscle through endothelium-dependent nitric oxide signalling	[160]
	Cornuside		Anti-inflammatory activity via the inhibition of NF-κB activity	[161]
	Loganic acid and cornuside		Increase in PPAR-α levels with reduced atherosclerotic plaque formation in cardiovascular diseases	[164]
**Neuroprotective effects**				
	Fresh fruits	Poland	Protection of the brain tissue by reducing the free radical content by increased activity of CAT and PON	[173]
**Antimicrobial activity**				
	n-Hexane extract of fresh fruits	Russian	Antibacterial activity against *S. aureus* and *E. coli*	[179]
	Methanol and ethanol extracts of fresh fruits	Poland	Antibacterial activity against *S. aureus*, *E. coli P. aeruginosa*	[180]
	Hydroalcoholic extract of fresh fruits	Serbia	Antibacterial activity against L. monocytogenes, B. cereus, *S. aureus*, *S. lutea*, *P. vulgaris*, *S. sonnei*, and *S. enteritidis*	[49]
**Cytotoxic activity**				
	Hydroalcoholic extract of dried fruits	Iran	Cytotoxic activity against MCF-7, PC-3, SKOV-3 and A549 cells	[186]
**Hepatoprotective effect**				
	Hydroalcoholic extract of dried fruits	Iran	Amelioration of AST, ALT and ALP levels	[188]
**Renal protective activity**				
	Hydroalcoholic extract of dried fruits	Iran	Renal protective effects via improving renal lesions, antioxidant enzymes, creatinine, uric acid and serum urea levels	[189]
**Cardioprotective and antiplatelet activities**				
	Hydroalcoholic extract of dried fruits	Iran	Cardioprotective effects through a reduction in PDW, attenuating myocardial lipid peroxidation level and recovering enzymatic defence system by increasing the levels of SOD, CAT, GPx, and modulating the bioenergetics state of cardiac tissue	[190,192]
	Loganin		Inhibition of angiotensin II. Cardioprotective effects through a decrease in pro-inflammatory cytokine secretion, suppression of phosphorylation of critical proteins including STAT3, JAK2, IκBα, and p65, and attenuation of cardiac fibrosis	[193,194,195,196,197]
**Ophthalmic activity**				
	Loganic acid		Reduction in nitric oxide with consequent decrease in IOP of the eye, ameliorating glaucoma	[82,198,199]
***C. sanguinea***				
**Antioxidant activity**	Hydroalcoholic extract of dried fruits	Turkey	Antioxidant activity in DPPH assay (IC_50_ of 1.205 mg/mL) and high inhibition against H_2_O_2_ activity (69.03%), while in Fe^2+^ chelating assay showed chelating activity of 51.24%	[127]
	Acidic methanol extract of dried fruits	Iran	Antioxidant activity in DPPH assay (IC_50_ of 94.83 μg/mL)	[123]
	Aqueous, methanol, acetone, ethyl acetate and petroleum ether extracts of fresh fruits	Serbia	Antioxidant activity in DPPH assay (IC_50_ of 358.59, 384.45, 537.83, 247.83, and 1202.85 μg/mL for methanol, aqueous, ethyl acetate, acetone, petroleum ether extracts, respectively)	[129]
***C. officinalis***				
**Antioxidant activity**				
	Hexane, chloroform, ethylacetate, ethanol, and aqueous extracts of dried fruits	Korea	Antioxidant activity in DPPH and *β*-carotene bleaching tests. Protection by ethanol extract of HUVECs from H_2_O_2_-initiated cell death	[130]
	Aqueous extract of fresh fruits	Korea	In diabetic rats, the activities of XO, CAT, and GST were lower than diabetic group	[131]
	Juice	Turkey	DPPH radicals scavenging activity, reducing power and oxygen radical absorbance capacity (22.31 μmole trolox equivalent/mL)	[102]
	Hydroalcoholic extract of fresh fruits	Korea	Antioxidant activity in DPPH test (IC_50_ of 99.32 μg/mL) and in ABTS test (40.7%), reduction in ferric complex (241.5 mM)	[133]
**Antidiabetic and anti-obesity activities**				
	Alcoholic extract of fruits	China	Increase in GLUT4 mRNA and protein expression with an increase in insulin production and accelerated glucose metabolism	[136]
	Fresh fruits	Japan	Reduction in proteinuria, hyperglycaemia, renal AGE formation, and the expression of related proteins, such as the receptor for AGEs, NF-κB, transforming growth factor-β1, and Nε-(carboxymethyl)lysine	[138]
	Methanol extract of fruits		Increase in cell viability through insulin mimicked activity of PEPCK expression	[140]
	Hydroalcoholic extract of fresh fruits	China	Decrease in blood glucose, HDL, LDL, TG, creatinine, and serum albumin levels	[141]
	Cornusdiglycosides A−J		α-Glucosidase inhibition with IC_50_ values in the range 78.9–162.2 mM	[114]
	Cornusiridoid A−F		*α*-Glucosidase inhibition	[99]
	Total saponin extract		Amelioration of liver and pancreas damage, regulation of insulin receptor, phosphatidylinositol 3-kinase, glucose transporter 4, and protein kinase B-associated signalling pathways	[142]
**Hypolipidemic and anti-atherosclerotic properties**				
	Fresh fruits		Reduction in serum and hepatic TC levels and SREBP-2. Increase in PPAR-α levels	[167]
**Neuroprotective effects**				
	Aqueous extract of dried fruits	South Korea	Alleviation of oxidative stress, decrease in immobility time in the forced swim test, an increase in serotonin levels and reduction in corticosterone and *β*-endorphin levels	[170]
	Decoction of fresh fruits	China	Protective effects against degenerative disease through stimulation of the neurite extension, increasing the percentage of PC12	[172]
	Cornel iridoid glycoside		Amelioration of learning and memory impairment by down-regulating the expression of Aβ and full-length amyloid precursor protein, as well as decreasing the hyperphosphorylation of tau protein	[168,169]
	Morroniside		Enhanced NGF function and BDNF expression. Promotion of the expression of Wnt7a and inhibition of the APC expression	[176,177,178]
**Antimicrobial activity**				
	Decoction extract of fresh fruits	China	Antibacterial activity against *E.coli* O157:H7	[185]
**Cytotoxic activity**				
	Decoction extract of fresh fruits	China	Inhibited hepatocellular carcinoma cell and leukemic cell growth	[187]
**Cardioprotective activity**				
	Loganin		Cardioprotective effects through a decrease in pro-inflammatory cytokine secretion, the suppression of the phosphorylation of critical proteins including STAT3, JAK2, IκBα, and p65, and the attenuation of cardiac fibrosis	[193,194,195,196,197]
**Antiosteoporosis activity**				
	Extract fruits	Korea	Inhibited receptor activator of nuclear factor-κB ligand (RANKL)-mediated osteoclast differentiation and RANKL-induced phosphorylation of p38 and c-JUN N-terminal kinase. Suppression of the protein expression of c-Fos and NFATc1	[200]
	Morroniside		Increase in type II collagen levels and improvement of proteoglycan levels in cartilage matrix	[201]
**Immunomodulatory activity**				
	Aqueous extract of fresh fruits	Korea	Reduction in the incidence of infections or allergic illnesses through the stimulation of the innate immune system; in particular, decreased production of IL-5, eotaxin, and IgE	[202]
	Hydroalcoholic extract of dried fruits	China	Improvement of the non-specific immunity, specific humoral immunity and specific cellular immunity	[203]

## 6. Toxicity

*C. mas* has been analysed for its potential toxicity [102,189,205]. West et al. [102] investigated the acute toxicity of *C. mas* in both animal and human models. The authors found that 5 mL/kg bw of *C. mas* supplementation in rats for 14 days showed no adverse effects. The average lethal dose (LD_50_) value was <5200 mg/kg. In a human study, it was demonstrated that 100 g/day for 6 weeks of fresh *C. mas* fruit consumption did not show any toxicity [205]. Previously, Es Haghi et al. [189] showed that doses of a water extract of *C. mas* fruits in the range 100–1650 mg/kg for 2 weeks in mice showed no toxicity (LD_50_ value of 1270 mg/kg). Toxicity studies have shown *C. mas* consumption is safe and has no side effects; however, high doses and long-term toxicity evaluations are required.

## 7. Conclusions

Since ancient times, fruits from the *Cornus* genus have been used in traditional medicine for their beneficial health properties. Some species are edible, particularly the European *C. mas* and *C. sanguinea*. *C. officinalis*, *C. kousa* and *C. controversa* are the edible species diffused in the Asiatic continent.

*C. mas* and *C. officinalis* are extensively studied plants with a high biological value, which is mainly connected to their polyphenols and iridoid content.

In particular, *C. mas* fruits are well-known as traditional components of jams, liquors, and other fruit-based products and are a rich source of bioactive molecules. Significant amounts of anthocyanins, flavonoids, and iridoids were identified in the fruits. These phytochemicals are linked with remarkable antioxidant, anti-inflammatory, and cancer properties. Additionally, *C. officinalis* exhibits a broad range of pharmacological activities, including anti-inflammatory, antioxidant, antiaging, cardioprotective, neuroprotective, and antibacterial effects. As described in our review, iridoids, flavonoids, terpenoids, polysaccharides, organic acids, and other compounds have been isolated and identified from *C. officinalis* fruits. This interest has recently prompted researchers to investigate also interspecific hybrids of *C. mas* × *C. officinalis* [206]. These hybrids are characterized by more iridoids than *C. mas* and more anthocyanins than *C. officinalis*.

Few phytochemical studies are present for other edible *Cornus* species. According to this review, the fruits of these *Cornus* species are rich in bioactive compounds, such as flavonoids, iridoids, anthocyanins, carotenoids, and other secondary metabolites; thus, their use might represent some benefits to human health.

Fruit extracts could effectively counter the onset of various illnesses linked to diabetes, obesity, and kidney and gastrointestinal disorders. However, exhaustive future studies of *C. sanguinea* and *C. controversa* fruits’ biological properties and chemical composition are still required. In addition, pharmacokinetic and pharmacodynamic studies on *C. mas* and *C. officinalis* fruits and their derived bioactive metabolites are necessary to confirm their effectiveness for human use. Moreover, toxicity studies on phytocomplex and single bioactive compounds are necessary to ensure the safety of these uses.

## Figures and Tables

**Table 1:** Traditional uses of *Cornus* fruits species.

	Traditional Use	Country	Reference
*C. mas*	Immune system strengthening	Serbia	[21]
	Fever	Albania	[22]
		Iran	[17]
		Slovakia	[15]
		Romania	[23]
	Tuberculosis digestive	Greece	[24]
	Cholera	Armenia	[25]
	Measles, chicken pox	Azerbaijan, Russia	[12,14]
	Vermifuge	Romania	[23]
	Malaria	Iran	[17]
	Cancer	Iran	[17]
	Headache	Croatia	[22]
	Sore throat	Azerbaijan, Russia	[12,14]
	Colds and flu	Turkey	[26]
	Asthmatic problems	Albania	[22]
	Cough	Turkey	[27]
	Bronchitis	Turkey	[28]
	Gastrointestinal disorders and inflammation	Turkey	[18]
		Greece	[24]
		Slovakia	[15]
		Albania	[22]
		Serbia	[21]
		Azerbaijan, Russia	[12,14]
	Bowel disease	Iran	[17]
	Stomach ulcers and colitis	Iran, Azerbaijan, Armenia, Georgia and Turkey	[12,13,14]
	Dyspepsia and colitis	Italy	[29]
	Diarrhoea	Iran	[17]
		Serbia	[30,31]
		Romania	[23]
		Azerbaijan	[32]
		Bosnia-Herzegovina	[33]
	Laxative	Serbia	[30]
		Turkey	[34]
	Urinary inflammation	Iran	[17]
		Turkey	[26]
	Excessive urination	USA	[35]
	Kidney function	China	[36]
	Kidney infection	Iran	[17]
	Kidney stones	Albania	[37]
		Iran	[17]
	Sweating	USA	[35]
	Wound healing	Iran, Azerbaijan, Armenia, Georgia and Turkey	[12,13,14]
	Skin diseases	Greece	[24]
		Bosnia-Herzegovina	[33]
	Sunstroke	Iran	[17]
	Bruises	Croatia	[22]
	Anaemia	Kosova	[38]
		Greece	[24]
		Serbia	[21]
		Azerbaijan, Russia	[12,14]
	Blood circulation	Kosova	[38]
	Menstrual bleeding	USA	[35]
		Greece	[24]
		Bosnia-Herzegovina	[33]
		Kosova	[38]
		China	[39]
	Rheumatism	Kosova	[38]
		Albania	[37]
	Rickets	Azerbaijan, Russia	[12,14]
	Gout	Greece	[24]
	Appetizer	Italy	[40]
	Obesity	Croatia	[22]
	Diabetes	Ukraine, Russia	[41]
	Cosmetics to exert favourable human complexion	Italy	[42]
*C. sanguinea*	Diarrhoea	Turkey	[19]
	Gastrointestinal disorders	Turkey	[20]
	Stomachaches, sore eyes	Turkey	[20]
	Sore throats	Turkey	[20]
	Astringent	Italy	[40]
*C. officinalis*	Dizziness	China	[43]
	Glaucoma	China	[44]
	Cataract	China	[44]
	Tinnitus	China	[43,44]
	Sore throat	China	[44]
	Astringent	China	[44]
	Cough	China	[44]
	Asthmatic problems	China	[44]
	Strengthening spleen and kidney	China	[44]
	Excessive urination and polydipsia	China	[9]
	Chronic nephritis	China	[44]
	Kidney and liver function, tonic	China	[8]
	Menstrual bleeding	China	[45]
	Diabetes	China	[44]
	Weakness of the waist, knees	China	[43]
	Arresting seminal emission	China	[44]
	Impotence	China	[44]
	Spermatorrhoea	China	[9]
	Night sweats	China	[44]
	Threatened abortion	China	[44]
*C. controversa*	Astringent Tonic	Korea and China	[46]
*C. kousa*	Diarrhoea Haemostatic agent	Korea Korea	[47] [47]

**Table 2:** The main flavonoids and proanthocyanidins of *Cornus* fruits.

Chemical Constituent	*C. mas*	*C. sanguinea*	*C. officinalis*	*C. kousa*	Reference
**Flavonoids**					
Ampelopsin 3-*O*-glucoside		✓			[58]
Aromadendrin	✓				[63]
Aromadendrin 7-*O*-glucoside	✓				[50]
4-Acetoxy-5,2′,4′,6′-*β*-pentahydroxy-3-methoxychalcone	✓				[63]
Catechin	✓				[49,50]
Epicatechin	✓				[49]
Epicatechin 3-*O*-gallate			✓		[60]
7,3′-dihydroxy-5,4′-dimethoxyflavanone	✓				[63]
Isorhamnetin 3-*O*-glucuronide		✓			[58]
Isorhamnetin hexoside				✓	[57]
Kaempferide			✓		[60]
Kaempferol			✓	✓	[59,62]
Kaempferol 3-*O*-galactoside	✓		✓		[50,64]
Kaempferol 3-*O*-glucoside	✓		✓	✓	[49,50,59,61,62]
Kaempferol 3-*O*-rhamnoside				✓	[61]
Kaempferol 3-*O*-rutinoside			✓		[8]
Myricetin	✓				[65]
Myricetin 3-galactoside	✓				[66]
Myricetin 3-*O*-rhamnoside				✓	[61]
Myricetin 3-*O*-*α*-L-arabinopyranoside-4′-*O*-*β*-D-glucopyranoside		✓			[58]
Naringenin			✓		[59]
Naringenin 3-*O*-methyl ester	✓				[65]
Isoquercitrin			✓		[60]
Quercetin	✓		✓		[59,67]
Quercetin 3-*O*-galactoside (hyperoside)	✓	✓	✓	✓	[20,50,57,58,60,62,64]
Quercetin 3-*O*-(6″-acetyl)galactoside (methyl ester)			✓	✓	[8,57]
Quercetin 3-*O*-galactopyranoside 4′-*O*-glucopyranoside	✓	✓	✓	✓	[20,49,50,57,58,64]
Quercetin 3-*O*-glucoside	✓		✓	✓	[8,50,62]
Quercetin 3-*O*-(6″-acetyl)glucoside				✓	[57]
Quercetin 3-*O*-(6″-acetyl)hexoside				✓	[57]
Quercetin 3,4′-di-*O*-glucoside		✓			[58]
Quercetin 3-*O*-glucuronide (querciturone)	✓	✓	✓		[20,49,50,58,60]
Quercetin 3-*O*-glucuronide methyl ester			✓		[64]
Quercetin 3-*O*-(6″-n-butyl glucuronide)			✓		[60]
Quercetin-3-*O*-(6″-malonyl)hexoside 1 and 2				✓	[57]
Quercetin 3-*O*-rhamnoside	✓	✓			[20,50]
Quercetin 3-*O*-rhamnosyl-(1→6)-galactopyranoside			✓		[64]
Quercetin 3-*O*-robinobioside	✓				[65]
Quercetin 3-*O*-rutinoside (rutin)	✓	✓	✓		[20,49,50,58,64]
Quercetin 3-*O*-xyloside	✓			✓	[50,57]
**Proanthocyanidins**					
Epicatechin- 4,8-epicatechin (Procyanidin B_2_)	✓				[49]

**Table 3:** The main anthocyanins are identified in *Cornus* fruits.

Chemical Constituent	*C. mas*	*C. sanguinea*	*C. officinalis*	*C. controversa*	*C. kousa*	Reference
Cyanidin 3-*O*-galactoside	✓		✓	✓	✓	[2,11,50,57,64,66,68,69,70]
Cyanidin 3-*O*-glucoside	✓	✓		✓	✓	[11,50,57,66,68,69,70,71]
Cyanidin 3-*O*-robinobioside	✓					[50,66,68,69,70]
Cyanidin 3-*O*-rutinoside	✓					[50,66,68,69,70]
Delphinidin-3-*O*-galactoside	✓		✓	✓		[2,11,50,64,66,68,69,70]
Delphinidin 3-*O*-glucoside	✓			✓	✓	[2,11,57,64,70]
Delphinidin 3-*O*-rutinoside				✓		[11]
Pelargonidin 3-*O*-galactoside	✓		✓	✓	✓	[2,11,50,57,64,66,68,69,70]
Pelargonidin 3-*O*-glucoside	✓					[50,66,68,69,70]
Pelargonidin 3-*O*-robinobioside	✓					[50,66,68,69,70]
Pelargonidin 3-*O*-rutinoside	✓					[50,66,68,69,70]
Peonidin 3-*O*-glucoside	✓					[50,66,68,69,70]
Petunidin 3-*O*-glucoside	✓					[66]

**Table 4:** The main lignans of *Cornus* fruits.

Chemical Constituent	*C. officinalis*	*C. kousa*	Reference
(7*S*,8*R*)- Dihydrodehydrodiconiferyl alcohol	✓	✓	[51,55]
(7*S*,8*R*)-5-Methoxydihydrodehydroconiferyl alcohol	✓		[51]
(7*S*,8*R*)-Urolignoside= (7S,8R)-dihydrodehydrodiconiferyl alcohol-4-*O*-*β*-D-glucopyranoside	✓		[51]
(7*S*,8*R*)-Dihydrodehydrodiconiferyl alcohol-9-*β*-D-glucopyranoside	✓		[51]
(7*S*,8*R*)-Dihydrodehydrodiconiferyl alcohol-9′-*β*-D-glucopyranoside	✓		[51]
(-)-Balanophonin		✓	[56]
Cornuskoside A = (7′*S*, 8′*R*)-dihydrodehydrodiconiferyl alcohol-4′-*O*-*β*-D-xylopyranoside,		✓	[56]
Officinalignan A = (7*S*,8*R*)-4,3′,9′-trihydroxyl-3,3′-dimethoxyl-7,8- dihydrobenzofuran-1′-propylneolignan-9-*O*-(6-*O*-galloyl)-*β*-D-glucopyranoside	✓		[51]
d-Pinoresinol	✓		[51]
Medioresinol	✓		[51]
Syringaresinol	✓		[51]
(+)-Pinoresinol		✓	[55]
Pinoresinol *O*-*β*-D-glucopyranoside	✓		[51]
Epi-pinoresinol	✓		[51]
Epi-syringaresinol	✓		[51]
(+)-Lariciresinol		✓	[55,72]
(+)-Isolariciresinol	✓		[51]
Isolariciresinol 9-*O*-*β*-D-glucopyranoside	✓		[51]
Secoisolariciresinol 9-*O*-*β*-D-glucopyranoside	✓		[51]
Dimethyl 3,3′,4,4′-tetrahydroxy-*δ*-truxinate	✓		[51]
Officinalignan B	✓		[51]
Threo-Guaiacylglycerol-*β*-coniferyl aldehyde ether		✓	[55]
Erythreo-Guaiacylglycerol-*β*-coniferyl aldehyde ether		✓	[55]

**Table 5:** The main acids and phenolic acids of *Cornus* fruits.

Chemical Constituent	*C. mas*	*C. sanguinea*	*C. officinalis*	*C. kousa*	Reference
**Acids and esters**					
2-Butoxybutanedioic acid			✓		[64,84]
Citric acid	✓		✓	✓	[57,65,79,80,86]
Fumaric acid	✓			✓	[57,65,79,80]
Isocitric acid	✓				[65,79,80]
Dimethylmalate			✓		[64]
Maleic acid	✓				[65,79,80]
Malic acid	✓		✓	✓	[57,64,75]
Butyl malic acid			✓		[64]
Malonic acid	✓				[65,79,80]
Oxalic acid	✓			✓	[57,65,79,80]
3-Hydroxy-2,4-di-amino-pentanoic acid			✓		[64]
Methyl quinate			✓		[64]
Quinic acid	✓				[65,79,80]
Shikimic acid	✓			✓	[57,65,79,80]
Succinic acid	✓		✓		[65,79,80,86]
Tartaric acid	✓		✓		[64,65,79,80]
Arjunolic acid				✓	[47]
Arjunglucoside II			✓		[64]
Asiatic acid				✓	[47]
19-Hydroxyasiatic acid				✓	[47]
Betulinic acid			✓	✓	[47,64]
Corosolic acid				✓	[87]
Maslinic acid				✓	[87]
Oleanic acid				✓	[88]
Pimaric acid			✓		[89]
Tormentic acid				✓	[47]
Ursolic acid	✓		✓	✓	[47,69,90]
2*α*-Hydroxylursolic acid				✓	[87]
**Phenolic acids and esters**					
3,5-Dihydroxybenzoic acid			✓		[64]
2-*O*-(4-Hydroxybenzoyl)-2,4,6-trihydroxyphenyl-methylacetate			✓		[64]
Caffeic acid	✓		✓		[64,75]
4-Caffeoylquinic acid				✓	[57]
Caffeoyltartaric acid dimethyl ester			✓		[85]
Caftaric acid monomethylester			✓		[60]
Chlorogenic acid	✓			✓	[49,52,57,82]
Neochlorogenic acid	✓	✓		✓	[49,52,57,71,82,91]
*p*-Hydroxycinnamic acid			✓		[64]
Coroffester A-D			✓		[92]
*p*-Coumaric acid	✓		✓		[64,81]
Ellagic acid	✓		✓		[49,52,64]
Ferulic acid	✓				[53]
Gallic acid	✓				[49,52]
Methyl gallate			✓		[64]
3,5-Dihydroxy-2-(2-methoxy-2-oxoethyl) phenyl 4-hydroxybenzoate			✓		[8]
Protocatechuic acid	✓		✓		[64,75]
Rosmarinic acid	✓				[75]
Salicylic acid	✓				[75]
Sinapic acid	✓				[75]
Syringic acid	✓				[75]
Vanillic acid	✓				[53]

**Table 6:** The main iridoids of *Cornus* fruits.

Chemical Constituent	Other Name	*C. officinalis*	*C. mas*	Reference
**Aglycons**				
Dehydro-morroniside aglycone		✓		[60,64]
(3*S*, 4*R*, 5*S*, 7*S*, 8*R,* 9*R*) Cornusfural A (*with a* 5*-hydroxymethylfurfural* *group at C-*3)		✓		[97]
(3*R*, 4*R*, 5*S*, 7*S*, 8*R,* 9*R*) Cornusfural B (*with* 5*-hydroxymethylfurfural group at C-*3)		✓		[97]
(1*R*, 3*R*, 4*R*, 5*S*, 7*S*, 8*S,* and 9*R*). Cornusfural C (*with* 5*-hydroxymethylfurfural group at C-*1 and *C-*3)		✓		[97]
**Other iridoids**				
Catalposide			✓	[67]
Cornuside		✓	✓	[8,52,60,82,98]
3′′,5′′-Dehydroxycornuside		✓		[99]
Demethoxycornuside		✓		[100]
Kingiside		✓		[60]
2′-*O*-*p*-Coumaroyl-kingiside		✓		[51,101]
Loganin		✓	✓	[8,52,82,98]
Loganin acid		✓	✓	[8,52,82,102]
7-*O*-Methylloganic acid		✓		[103]
Loganin-7-*O*-1′-malate	Logmalicid A	✓		[8]
Loganin-7-*O*-4′-malate	Logmalicid B	✓		[8]
2ʹ-*O*-*p*-Coumaroylloganin		✓		[51]
8-Epiloganin		✓		[104]
Secoxyloganin		✓		[8,60]
Secologanoside		✓		[60,100]
Secoxyloganin	Loniceroside [105]	✓		[100]
Sweroside		✓	✓	[52,82,106]
Swertiamarin		✓		[107,108]
Verbenalin	Cornin *	✓		[64,101]
**Morronisides**				
7-*α*-Morroniside		✓		[105]
7-*β*-Morroniside		✓		[105]
7-*α*-*O*-methyl-morroniside		✓		[100]
7-*β-O*-Methyl-morroniside		✓		[64,100,105]
7-*α-O*-Ethyl-morroniside		✓		[64]
7-*β-O*-Ethyl-morroniside		✓		[64]
7-*O*-Butyl-morroniside		✓		[64,109]
7-*β-O*-Dimethyl butanedioate morroniside		✓		[85]
7-*α-O*-Ethyl-4′,6′-*O*-(2′″hydroxymethylfuran 5″methylidene)-morroniside	Cornusfuroside A	✓		[110]
7-*β-O*-Ethyl-4′,6′-*O*-(2′″hydroxymethylfuran 5″methylidene)-morroniside	Cornusfuroside B (isomer 1″S)	✓		[110]
7-*β-O*-Ethyl-4′,6′-*O*-(2′″hydroxymethylfuran-5″methylidene)-morroniside	Cornusfuroside C(isomer 1″R)	✓		[110]
7-*β-O*-(5″Methylfurfural)-4′,6′-*O*-(2′″hydroxymethylfuran-5″methylidene)-morroniside	Cornusfuroside D(1″R)	✓		[110]
7-*β-O*-(5″Methylfurfural)-6′*-O*-(phenyllactic acid)-morroniside	Cornusphenoside A	✓		[111]
7-*α*-*O*-(5″Methylfurfural)-6′*-O*-(phenyllactic acid)-morroniside	Cornusphenoside B	✓		[111]
6′*-O*-(Phenyllactic acid)-7-*β-O*-methyl-morroniside	Cornusphenoside C	✓		[111]
6′*-O*-(Phenyllactic acid)-7- *α-O*-methyl-morroniside	Cornusphenoside D	✓		[111]
7-*β*-*O*-(*p*-Hydroxyphenyl) propyl-1- *α-*morroniside	Cornusphenoside E	✓		[112]
7-*β-O*-(*p*-Hydroxyphenyl) ethyl- morroniside	Cornusphenoside F	✓		[112]
7-*α-O*-(*p*-Hydroxyphenyl) ethyl-morroniside	Cornusphenoside G	✓		[112]
7- *β-O*-(2′Hydroxymethyl-5′methylfuran)-morroniside	Cornusphenoside H	✓		[112]
7-*β-O*-(5′Methylfurfural)-morroniside	Cornusphenoside I	✓		[112]
**Morronisides glycosides**				
Methylquinate(1′-*O*-7-*α*)-morroniside	Cornusglucoside A	✓		[113]
Glycerol-(1′-*O*-7-*α*)-morroniside	Cornusglucoside B	✓		[113]
**Diglycosides**				
*β*-D-Fructofuranosyl-(6″-*O*-7-*β*)-morroniside	Cornusdiglycoside A	✓		[114]
*β*-D-Fructofuranosyl-(6″-*O*-7-*α*) morroniside	Cornusdiglycoside B	✓		[114]
*β*-D-Fructofuranosyl-(1″-*O*-7-*α*) morroniside	Cornusdiglycoside C	✓		[114]
*α*-D-Fructofuranosyl-(6″-*O*-7-*β*)-morroniside	Cornusdiglycoside D	✓		[114]
(6′-*O*-2″)- Fructopyranosyl-7*α*-*O*-methylmorroniside	Cornusdiglycoside E	✓		[114]
(6′-*O*-2″)- Fructopyranosyl-7*β*-*O*-methylmorroniside	Cornusdiglycoside F	✓		[114]
*α*-D-Glucopyranosyl-(1″-*O*-7-*α*)-morroniside	Cornusdiglycoside G	✓		[114]
*α*-D-Glucopyranosyl-(1″-*O*-7-*β*)-morroniside	Cornusdiglycoside H	✓		[114]
*α*-D-Glucopyranosyl-(2″-*O*-7-*β*)-morroniside	Cornusdiglycoside I	✓		[114]
*β*-D-Glucopyranosyl-(2″-*O*-7-*β*)-morroniside	Cornusdiglycoside I	✓		[114]
*α*-D-Glucopyranosyl-(6″-*O*-7-*β*)-morroniside	Cornusdiglycoside J	✓		[114]
*β*-D-Glucopyranosyl-(6″-*O*-7-*β*)-morroniside	Cornusdiglycoside J	✓		[114]
**Dimers (morroniside-morroniside)**				
7*β*-O-Methylmorroniside-(6′-O-7″)-*α*-morroniside	Cornuside J	✓		[104]
7*β*-*O*-Methylmorroniside-(6′-*O*-7″)-*β*-morroniside	Cornuside A	✓		[104]
7*α*-*O*-Methylmorroniside-(6′-*O*-7″)-*β*-morroniside	Cornuside E	✓		[104]
7*α*-*O*-Methylmorroniside-(6′-*O*-7″)-*α*-morroniside	Cornuside G	✓		[104]
7*β*-*O*-Methylmorroniside-(2′-*O*-7″)-*α*-morroniside	Cornuside I	✓		[104]
7*β*-*O*-Methylmorroniside-(2′-*O*-7″)-*β*-morroniside	Cornuside B	✓		[104]
7*α*-*O*-Methylmorroniside-(2′-*O*-7″)-*α*-morroniside	Cornuside H	✓		[104]
7*β*-*O*-Methylmorroniside-(3′-*O*-7″)-*β*-morroniside	Cornuside C	✓		[104]
7*α*-*O*-Methylmorroniside-(3′-*O*-7″)-*β*-morroniside	Cornuside F	✓		[104]
7*β*-*O*-Methylmorroniside-(4′-*O*-7″)-*β*-morroniside	Cornuside D	✓		[104]
7*α*-*O*-Ethylmorroniside-(6′-*O*-7″)-*β*-morroniside	Cornuside K	✓		[104]
7*β*-*O*-Ethylmorroniside-(6′-*O*-7′′)-*β*-morroniside	Williamsoside D	✓		[104]
**Dimers (cornuside-morroniside)**				
Cornuside-(2′-*O*- 7′′′)-*β*-morroniside	Cornusdiridoid A	✓		[99]
Cornuside-(2′-*O*-7′′′)-*α*-morroniside	Cornusdiridoid B	✓		[99]
Cornuside-(3′-*O*-7′′′)-*α*-morroniside	Cornusdiridoid C	✓		[99]
Cornuside-(4′-*O*-7′′′)-*α*-morroniside	Cornusdiridoid D	✓		[99]
Cornuside-(6-*O*-7′′′)-*β*-morroniside	Cornusdiridoid E	✓		[99]
Cornuside-(6′-*O*-7′′′)-*α*-morroniside	Cornusdiridoid F	✓		[99]
**Dimers (loganin-morroniside)**				
Loganin-(6′-*O*-7″)-*α*-morroniside	Cornuside L	✓		[104]
Loganin-(2′-*O*-7″)-*α*-morroniside	Cornuside M	✓		[104]
Loganin-(4′-*O*-7″)-*β*-morroniside	Cornuside N	✓		[104]
Loganin-(4′-*O*-7″)-*α*-morroniside		✓		[51,105]
Loganin-(7-*O*-7″)-*β*-morroniside	Cornuside O	✓		[104]
**Monoterpene indol alkaloid from *C. officinalis***				
3*β*(*R*)-Vincosamide		✓		[99]
7-epi-Javaniside		✓		[99]
Javaniside		✓		[99]

* also identified in *C. kousa* [61].
